# Differential Proteome Analysis of Human Neuroblastoma Xenograft Primary Tumors and Matched Spontaneous Distant Metastases

**DOI:** 10.1038/s41598-018-32236-1

**Published:** 2018-09-18

**Authors:** Lorena Hänel, Tobias Gosau, Hanna Maar, Ursula Valentiner, Udo Schumacher, Kristoffer Riecken, Sabine Windhorst, Nils-Owe Hansen, Laura Heikaus, Marcus Wurlitzer, Ingo Nolte, Hartmut Schlüter, Tobias Lange

**Affiliations:** 1 Institute of Clinical Chemistry and Laboratory Medicine, University Medical Center Hamburg-Eppendorf, Martinistrasse 52, 20246 Hamburg, Germany; 2 Institute of Anatomy and Experimental Morphology, University Cancer Center Hamburg, University Medical Center Hamburg-Eppendorf, Martinistrasse 52, 20246 Hamburg, Germany; 3 Research Department Cell and Gene Therapy, Clinic for Stem Cell Transplantation, University Medical Center Hamburg-Eppendorf, Martinistrasse 52, 20246 Hamburg, Germany; 4 Department of Biochemistry and Signal Transduction, University Medical Center Hamburg-Eppendorf, Martinistrasse 52, 20246 Hamburg, Germany; 5 Max Planck Institute for the Structure and Dynamics of Matter, Luruper Chaussee 175, 22761 Hamburg, Germany; 6 Small Animal Clinic, University of Veterinary Medicine Hannover, Bünteweg 9, 30559 Hannover, Germany

## Abstract

Metastasis formation is the major cause for cancer-related deaths and the underlying mechanisms remain poorly understood. In this study we describe spontaneous metastasis xenograft mouse models of human neuroblastoma used for unbiased identification of metastasis-related proteins by applying an infrared laser (IR) for sampling primary tumor and metastatic tissues, followed by mass spectrometric proteome analysis. IR aerosol samples were obtained from ovarian and liver metastases, which were indicated by bioluminescence imaging (BLI), and matched subcutaneous primary tumors. Corresponding histology proved the human origin of metastatic lesions. Ovarian metastases were commonly larger than liver metastases indicating differential outgrowth capacities. Among ~1,900 proteins identified at each of the three sites, 55 proteins were differentially regulated in ovarian metastases while 312 proteins were regulated in liver metastases. There was an overlap of 21 and 7 proteins up- and down-regulated at both metastatic sites, respectively, most of which were so far not related to metastasis such as LYPLA2, EIF4B, DPY30, LGALS7, PRPH, and NEFM. Moreover, we established *in vitro* sublines from primary tumor and metastases and demonstrate differences in cellular protrusions, migratory/invasive potential and glycosylation. Summarized, this work identified several novel putative drivers of metastasis formation that are tempting candidates for future functional studies.

## Introduction

The most life-threatening, but least understood aspect of cancer biology is the formation of distant metastases, which account for 90% of cancer-related deaths^1^. In particular, more than 50% of neuroblastoma (NB) patients present wide-spread metastasis at diagnosis^2^. In order to improve therapy, it is therefore of utmost importance to improve our understanding of the pathogenesis of metastasis formation. Very few studies have compared the molecular characteristics of single NB metastases with that of primary tumors^3^, and none studied multiple metastases from single individuals. Therefore, suitable *in vivo* animal models of metastatic tumors and matched primary tumors are needed to study the molecular mechanisms driving metastasis. The development of such models has been the subject of a range of previous studies, all of which aim to address the scientific question of how metastases occur. During the past decades, three key principles have emerged that should be considered for modeling metastasis formation *in vivo*:

(I) Only very few primary tumor cells will form distant metastases. These future metastatic cells are distinct from the surrounding primary tumor cells as they have loosened their cell-cell and cell-matrix contacts and hence acquired invasive capacity, *e.g*. through the cell biological program of epithelial-to-mesenchymal transition, EMT^4^. Upon detachment from the primary tumor and migration through the adjacent connective tissue, the future metastatic cells enter the bloodstream as so-called circulating tumor cells (CTC).
(II) Only very few CTCs will survive in the bloodstream until extravasation at a distant site. This is supposed to be mainly due to the adverse conditions existing in the bloodstream: blood flow-induced shear stress, intrinsic apoptosis (caused by the detachment from the extracellular matrix = *anoikis*), and immune cell attack mainly by natural killer cells^5^. CTC have to adhere to endothelial cells (EC) lining the vascular walls and to accomplish transendothelial migration in order to form disseminated tumor cells (DTC) at a distant site^6^.
(III) Only some of the DTC will actually form a clinically detectable metastasis. Single DTC can otherwise remain in a state of quiescence (dormancy) without producing clinical symptoms. Several pathways have been suggested to regulate dormancy or outgrowth of DTC in human cancer and it is widely assumed that the reversion of EMT, mesenchymal-to-epithelial transition (MET), promotes colonization^7^.

Importantly, several previous studies on the mechanisms of metastasis formation were either limited to *in vitro* assays of single aspects of the metastatic cascade or applied mouse models using intravenous (tail vein) or intracardiac (left ventricle) tumor cell injection^8–12^. These attempts, however, do not reflect the entire complexity of metastasis formation as outlined above as they circumvent, *e.g*., relevant steps at the primary tumor site. Therefore, we and others started to develop mouse models of spontaneous metastasis formation of human tumors, in which the single steps of the metastatic cascade are largely reflected^13–15^, and to characterize the tumor cells at the molecular (*e.g*., genomic) level at different metastatic stages^16^.

Most of the published metastasis studies are driven by a distinct hypothesis and therefore focused on one particular metastatic site or the functional analysis of single (groups of) molecules. The identification of novel drivers of metastasis formation, however, requires an unbiased screening approach such as differential mass spectrometric proteome analysis of tissues from different metastatic sites and corresponding primary tumors. However, proteome analysis is often hindered by a variable extent of enzymatic protein degradation during tissue collection and subsequent mechanical homogenization. This problem can be circumvented by the use of infrared (IR) lasers that convert tissues into aerosols containing all tissue molecules. In short, during irradiation of the tissue with the IR-laser, the tissue water molecules absorb the energy of the laser. A large part of the absorbed energy is converted into translational energy causing a kind of explosion of the tissue. Through fast vaporization and the heat transferred to the tissue, enzymatic degradation is reduced significantly. The IR-laser is meanwhile commonly applied in dermatology^17^, dentistry^18^, and otolaryngology^19^. However, this technique has not yet been used for tissue sampling for proteome analysis of xenograft primary tumors and matched spontaneous metastases.

On this background, the aim of this study was to establish spontaneous metastasis xenograft mouse models (resembling all major steps of metastasis formation including primary tumor growth) for the unbiased identification of metastasis-related proteins by sampling tissue by IR- laser ablation and subsequent proteome analysis. Moreover, we aimed to recover tumor cells from metastases and primary xenograft tumors for *in vitro* expansion and characterization.

## Materials And Methods

### Lentiviral transduction and cell culture

To enable simultaneous recognition of all metastatic sites in our planned xenograft models, the tumor cells had to be prepared for a whole body *in vivo*-imaging technique. For this purpose, the human NB cell line LAN-1 was used to generate LAN1-*Luc2/mCherry* cells expressing the luciferase from *Photinus pyralis* and the fluorescent protein mCherry. In short, the Luc2 cDNA (Addgene Plasmid #24337, Cambridge, USA) was cloned into the 3rd generation HIV1 derived SIN vector LeGO-iC2-Puro + expressing mCherry as marker gene and puromycin N-acetyl-transferase conferring puromycin resistance^20^. For lentiviral transduction of cancer cells, 1 × 10^5^ cells/mL were plated in 24 well plates and incubated for 24 h. Then supernatants containing viral particles and 8 µg/mL polybrene (Sigma, München, Germany) were added for 24 h. For the selection of transduced cancer cells, regular culture medium was supplemented with 2.5 µg/mL puromycin.

LAN-1-*Luc2/mCherry* cells were cultured under standard cell culture conditions (37 °C, 95% relative humidity, 5% CO2) in RPMI-1640 medium supplemented with 10% heat-inactivated fetal bovine serum, 2 mM glutamine, 100 U/mL penicillin and 100 µg/mL streptomycin (Gibco, Paisley, Scotland).

### Spontaneous metastasis xenograft mouse model

1 × 10^6^ LAN1-*Luc2/mCherry* cells were injected subcutaneously (s.c.) into the right flank of 12 weeks old female rag2^*−/−*^ mice (rag2 model 601, Taconic, Hudson, NY, USA) lacking mature B and T cells due to constitutive knockout of the *recombination activating gene 2*, which is essential for V(D)J rearrangement (n = 20). The tumor cells were suspended in 100 µl cell culture medium without supplements and mixed 1:2 with Matrigel (BD, Franklin Lakes, NJ, USA) immediately before injection. All mice were kept in individually ventilated cages under pathogen-free conditions, fed with sterile standard food and water *ad libitum*, and were regularly monitored concerning s.c. tumor growth. As soon as the primary tumors have grown up to about 1.5 cm^3^ or ulcerated the mouse skin, mice underwent BLI under inhalation anesthesia with isoflurane using the IVIS 200 (Perkin Elmer, Waltham, MA, USA) and were afterwards anesthetized by intraperitoneal injection of ketamine hydrochloride (100 mg/mL, 1.2 mL/kg; Graub, Bern, Switzerland) and xylacine hydrochloride (20 mg/mL; 0.8 mL/kg; Bayer, Leverkusen, Germany). The primary tumors were then surgically resected and immediately cryo-preserved in liquid nitrogen, or fixed in 4% formalin or recovered for *in vitro*-cultivation. Skin closure was performed with disposable skin staples (3 M Health Care, Borken, Germany) and mice received Carprofen s.c. (5 mg/mL, 5 mg/kg, Zoetis, Berlin, Germany) immediately after surgery as well as on the first and second post-operative day. All animal experiments were approved by the local animal experiment approval committee (Behörde für Gesundheit und Verbraucherschutz, Amt für Verbraucherschutz, Lebensmittelsicherheit und Veterinärwesen, assigned project No. 80/16), supervised by the institutional animal welfare officer and performed in accordance with relevant guidelines and regulations.

### Post-surgery monitoring of metastasis outgrowth by BLI

All mice were examined with BLI before and after excision of the primary tumor. To measure luminescence emission, luciferin (150 mg/kg, Sigma) was injected i.p. with a metabolic distribution period of at least 10 minutes *in vivo*. If one or several regions indicated metastases, the animal was sacrificed by cervical dislocation (13 to 41 days after primary tumor excision) to collect samples from affected visceral organs. After dissection of luminescent organs, the BLI signal was precisely localized by an *ex vivo* re-scan of the excised organ and metastatic tissue was (I) immediately cryo-preserved for subsequent laser ablation, (II) fixed in 4% formalin for histology or (III) recovered for *in vitro*-cultivation.

### Tissue homogenization with an IR-laser

**Figure 1:**
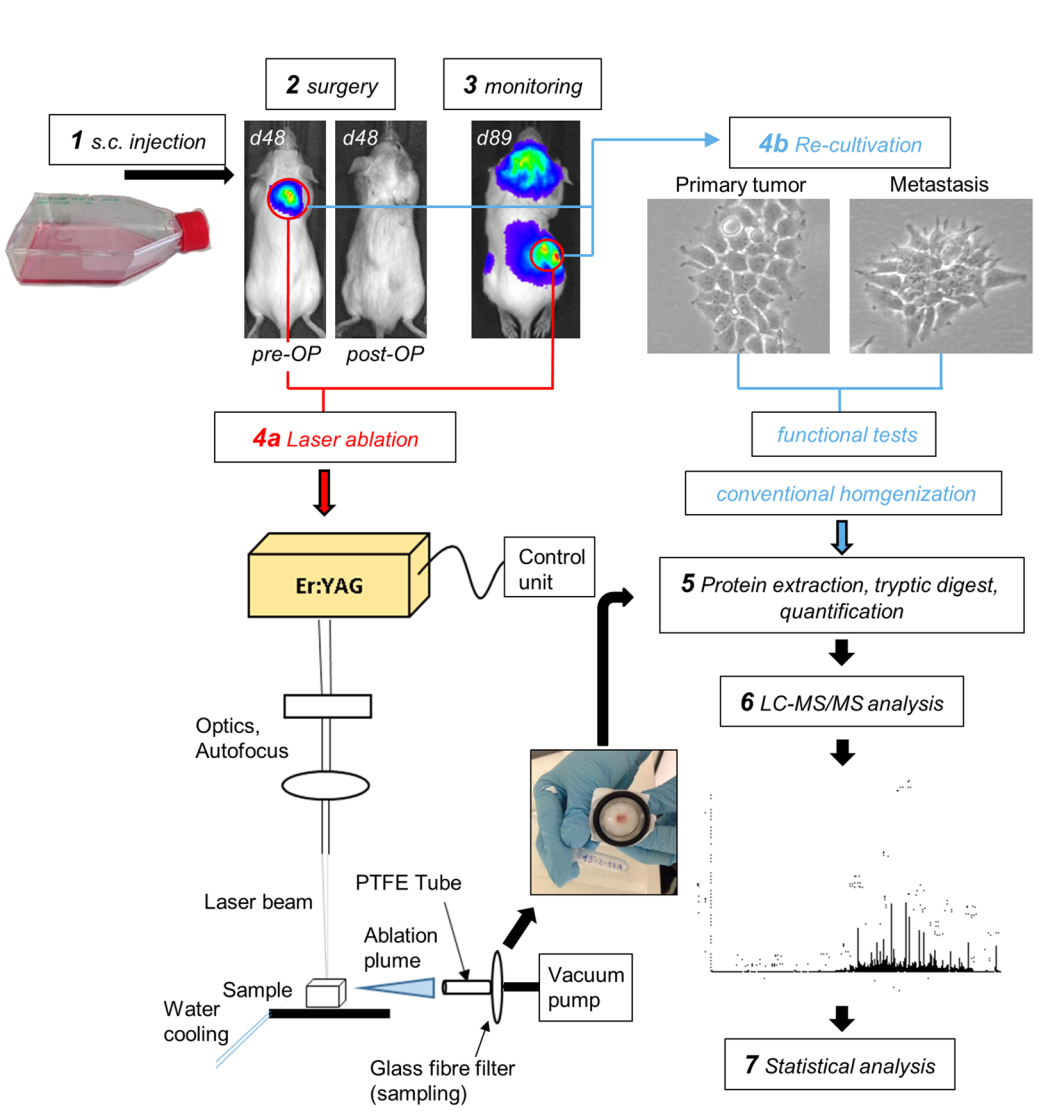
Experimental setup. Human neuroblastoma LAN-1-Luc2/mCherry cells were subcutaneously injected into immunodeficient *rag2*^*−/−*^ mice and surgically resected at a xenograft tumor size of ~1 cm^3^. Before and after surgery, mice were analyzed by bioluminescence imaging (BLI) to demonstrate the absence of detectable metastases at the time of surgery. Primary tumor cells were retrieved for *in vitro* expansion and establishment of the subline LAN-1-PT. Regular post-operative BLI scans were used to monitor the outgrowth of distant metastases. These lesions were either subjected to IR-laser ablation of proteins and subsequent proteome analysis or recovered for *in vitro*-cultivation. The resulting metastatic sublines LAN-1-Met-L and LAN-1-Met-O were functionally characterized and prepared for proteome analysis.

Cryo-preserved primary tumor or metastasis samples were ablated with an Er:YAG infrared laser (AdvErLEvo, J.Morita Mfg. Corp. Kyoto, Japan). To avoid enzymatic degradation of the proteins in the IR-laser ablated tissue homogenates, the aerosol plume, which was developing during ablation, had to be collected without thawing. Therefore, the cryo-preserved samples were mounted on a cooled plate at −10 °C and the IR-laser operating parameters were experimentally optimized for fast and homogenous vaporization without visible thermal damage. Likewise, chipping of big particles or material chunks was avoided. The pulse duration was 300 µs, energy per pulse set to 18 mJ and the repetition rate to 25 Hz. An autofocus system was installed to maintain the laser beam focused to a spot of about 250 µm diameter also on rough tissue surfaces. The energy setting and focal spot dimensions resulted in an average applied fluence of 37 J/cm^2^. The ablation time was set to 45 sec, scanning the laser beam on a square surface of 3 × 3 mm^2^, to achieve a near complete vaporization of the sample volume. During ablation, the aerosol was sucked in by a vacuum pump (Vacuubrand, CVC 2000, vacuum of 650 mbar, Wertheim, Germany) connected to a short 4 cm PTFE tubing system coupled to a home-built steel mounting, holding the glass fibre filter (Hahnemühle, Dassel, Germany, Grade GF 50, air permeability: 33 L/m²s) in place for sample collection. The setup enables to collect the ablation plume in seconds trapped as a dry condensate, ready to be prepared for mass spectrometric proteomics. Please see Fig. 1 for illustration of the experimental setup.

### Histology and immunohistochemistry

For histologic evaluation of primary tumor and metastasis tissues, samples were fixed for 48 h in 4% formalin, embedded in paraffin, cut into 4 µm thin sections and H&E-stained. Consecutive slides were used for immunohistochemistry against human NCAM (CD56): after de-paraffinization, antigen retrieval was performed with Dako S1699 Retrieval Solution at pH6 in a microwave for 2 × 4 min. Final concentration of the primary antibody (anti-NCAM, Leica #NCL-CD56-1B6) was 0.88 µg/mL and visualization of bound antibody was performed with the LSAB + Dako Real Detection System K5005. H&E- as well as anti-NCAM-stained slides were digitalized using a Mirax scanner (Zeiss, Jena, Germany). Photomicrographs were taken using Pannoramic viewer 1.15.2 SP 2 (3D Histech, Budapest, Hungary).

### Re-cultivation of primary tumor and metastasis samples

A small piece of surgically excised primary tumors as well as metastatic tissues from selected mice (ovarian and liver metastases) was minced with a razor blade and ground through a 100 µm mesh size filter (Corning cell strainer, VWR) stored in a 6-well containing standard cell culture medium. For pressing the tumor pieces through the cell strainer, a sterile syringe plunger was used. Afterwards, the filter was removed and the 6-well plate was incubated under standard conditions. Cell culture medium was replaced after 48 h for the first time and adherent cells were sub-cultivated depending on the tumor cell yield and proliferative behavior. The resulting *in vitro* sublines LAN-1-PT (derived from the primary tumor), LAN-1-Met-O (derived from an ovarian metastasis) and LAN-1-Met-L (derived from a liver metastasis) were subjected to functional analysis. Three replicates of each subline were thoroughly washed with PBS (Gibco, Paisley, Scotland) to avoid FCS contamination and dissolved in SDC buffer (Sodium Deoxycholate) for subsequent proteome analysis.

### Proteome analysis: Sample preparation

Tissue ablation products captured on the glass fiber filter (laser ablation products of metastasis and primary tumor tissues) were extracted using 1% sodium deoxycholate (SDC, extraction buffer) at pH 8 and 0.1 M triethylammonium bicarbonate (TEAB). The sampling filters were incubated with the extraction buffer on a shaker for 10 min at 95 °C, followed by 5 min ultrasonication (Bandelin, Sonoplus 2200, Berlin, Germany, 6 cycles, 30 seconds). Proteins from cultured cells were extracted by adding extraction buffer and incubation on a shaker for 5 min at 95 °C, also followed by 5 min ultrasonication. Protein quantification was performed with a bichinonic acid assay (BCA) performed in accordance with the literature^21^ (Tecan Spark, Männedorf, Switzerland). Tryptic digestion of 50 µg protein extract of each sample was performed in a 10 kDa microcentrifuge filter (Merck, Darmstadt, Germany) in accordance with Wisniewski^22^. Briefly, 2 × 500 µL of 6 M urea were added to the FASP filter and removed by centrifugation. Reduction and alkylation were performed adding 100 mM dithiothreitol (DTT) for 10 min at 56 °C followed by 300 mM iodacetamide (IAA) for 30 min in the dark. 425 µL of 100 mM NH_4_HCO_3_ followed by 0.5 µg trypsin were added to the sample and incubated for 16 hours at 37 °C. Tryptic digestion was stopped by centrifugation at 14000 × g for 20 minutes. The obtained digest was evaporated to complete dryness with a vacuum concentrator (SpeedVac™, Thermo scientific, Bremen) and stored at −20 °C.

HPLC-grade water, methanol and acetonitrile were obtained from Merck (Darmstadt, Germany). Sequence-grade trypsin and resuspension buffer were purchased from Promega (Madison, USA). Urea, DTT, IAA and NH_4_HCO_3_ were obtained from Sigma-Aldrich (Munich, Germany). TEAB and the BCA Kit were preserved from Thermo Scientific (Bremen, Germany), and formic acid (FA) from Honeywell Fluka (Bucharest, Romania).

### Proteome analysis: Liquid-chromatography coupled to tandem mass spectrometry (LC-MS/MS)

Tryptic peptides were resuspended in 50 µL of 0.1% FA and analyzed by LC-MS/MS on a nano-liquid ultra-pressure chromatography system (Dionex ultimate 3000 RSLCnano, Thermo scientific, Bremen, Germany) coupled to a linear trap quadrupole orbitrap tandem mass spectrometer (Orbitrap Fusion, Thermo scientific, Bremen, Germany) via nano-electrospray ionization-source (NSI). 1 µl of each sample was loaded onto a trapping column (Symetry C18 Trap Column; 100 Å, 5 μm, 180 μm × 20 mm, Waters, Eschborn, Germany) and washed with 3% buffer B (99.9% Acetonitrile, 0.1% FA) for 5 min. Then the peptides were eluted onto a reversed phase capillary column (Acclaim PepMap RSLC, 75 µm × 500 mm, C18, 2 µm, 100 Å, Thermo scientific, Bremen, Germany) and separated through a gradient from 3 to 28% buffer B in 35 min and from 28 to 35% in 40 min (0.3 µl/min). Eluting peptides were ionized by NSI (I.D. 10 µm, New Objective, Woburn, USA) at a capillary voltage of 1800 V. The maximum injection time was 120 ms for an AGC target of 2e^5^. The m/z range at MS level was set from 400 to 1300 Da with a resolution of 120.000 FWHM at m/z 200 for data dependent acquisition. Data dependent acquisition was performed in Top Speed mode, the most intense precursor ions with intensity greater than 1e^4^ were selected for fragmentation with a normalized HCD collision energy of 30%. Fragment spectra were recorded with a maximum injection time of 60 ms in the ion trap. The mass spectrometry proteomics data have been deposited to the ProteomeXchange Consortium via the PRIDE partner^23^ repository with the dataset identifier PXD009744.

### Quantitative proteomics

Raw data from LC-MS analysis were processed with Max Quant (Version 1.5.8.3) (PMID 19029910), identification performed with Andromeda against the human SwissProt database (www.uniprot.org, downloaded February 10, 2018, 20303 entries), the murine SwissProt database (downloaded February 16, 2017, 16750 entries) and a contaminant database with 298 entries. The MaxQuant parameters were set as followed: the precursor mass tolerance was set to 10 ppm, the fragment mass tolerance was set to 0.5 Da and two missed cleavages were allowed for peptide identification; an FDR of 1% was given and a maximum of 5 modifications per peptide were allowed. As a fixed modification the carbamidomethylation on cysteine residues and as variable modifications the oxidation of methionine residues and the acetylation of protein N-terminals were set. The LFQ minimum ratio count was set to 1.

Perseus (Version 1.6.0.2) statistical analysis of LFQ intensities was performed with at least three replicates from every sample group. Proteins only identified by site, reversed hits, contaminants and proteins matching exclusively with the murine database were removed. At least two unique peptides had to be found to identify a protein correctly. All values were transformed with the logarithm of 10 and the median was subtracted. Two categorical groups were created and two values in at least one group had to be valid for further steps. Missing values were replaced from normal distribution and a two sample t-test was processed with an FDR of 0.05. Received significantly regulated proteins with and at least 2-fold change were considered for further analysis.

### Functional *in vitro* assays with re-cultivated primary tumor and metastasis samples

Within the first 10 passages after recovery from the mice, LAN-1-PT, LAN-1-Met-O and LAN-1-Met-L cells were subjected to different functional tests concerning single steps of the metastatic cascade. First, cellular protrusions of these sublines were investigated by staining F-actin with Alexa Fluor 488-conjugated phalloidin and the migratory and invasive behavior was compared using CorningFluoroBlok or CorningBioCoat transwell migration or invasion assays (8 µm pore size), respectively, according to the manufacturer’s instructions (Corning, VWR, Darmstadt, Germany). Moreover, colony formation assays were performed using soft agar. The initial cell count was 300 cells/mL and the number and diameter of spheroid and discoid colonies were determined four days after seeding using a light microscope. Finally, the capacity of the tumor cells to bind murine E- and P-selectin was determined using flow cytometry as described before^24^.

### Characterization of increased E-selectin ligands on the ovarian metastasis subline

The role of *O*-GalNAc-glycoproteins for rmE-selectin binding was investigated by adding 20 mM GalNAc-α-*O*-benzyl (Sigma) for 72 h to the standard tumor cell culture prior to the E-selectin binding assay. Likewise, tumor cell cultures were treated for 1 h with 10 mU/mL neuraminidase from *Clostridium perfringens* (Roche, Mannheim, Germany) or with 1 mg/mL pronase from *Streptomyces griseus* (Roche) under serum-free conditions at 37 °C for 1 h or 45 min before the E-selectin binding assay to analyze the role of sialic acid residues or glycoproteins, respectively. Detrimental effects of all treatments were excluded by propidium iodide staining immediately before flow cytometry. To test which linkage of sialic acid was mainly affected by neuraminidase, lectin binding flow cytometry was performed using biotinylated *Maackia amurensis* lectin II (MAA-II, detecting α-2,3-linked sialic acid) and *Sambucus nigra* agglutinin I (SNA-I, detecting α-2,6-linked sialic acid) (both from Vector Labs, Burlingame, USA). Both lectins were marked with streptavidin-APC (Sigma) before incubation with the tumor cells. To test for carbohydrate-specific binding, one tumor cell sample was pre-treated with periodic acid, which oxidizes free sialic acid residues and by this cracks the ring structure of the carbohydrate, which is required for the binding of MAA-II and SNA-I.

### qPCR profiler array human glycosylation

Glycosyltransferase expression levels were compared using RT^2^ human glycosylation qPCR profiler arrays (SA Biosciences, Qiagen) as described before^25^.

## Results

### Tumor growth and spontaneous metastasis formation in LAN-1 xenografts

**Figure 2:**
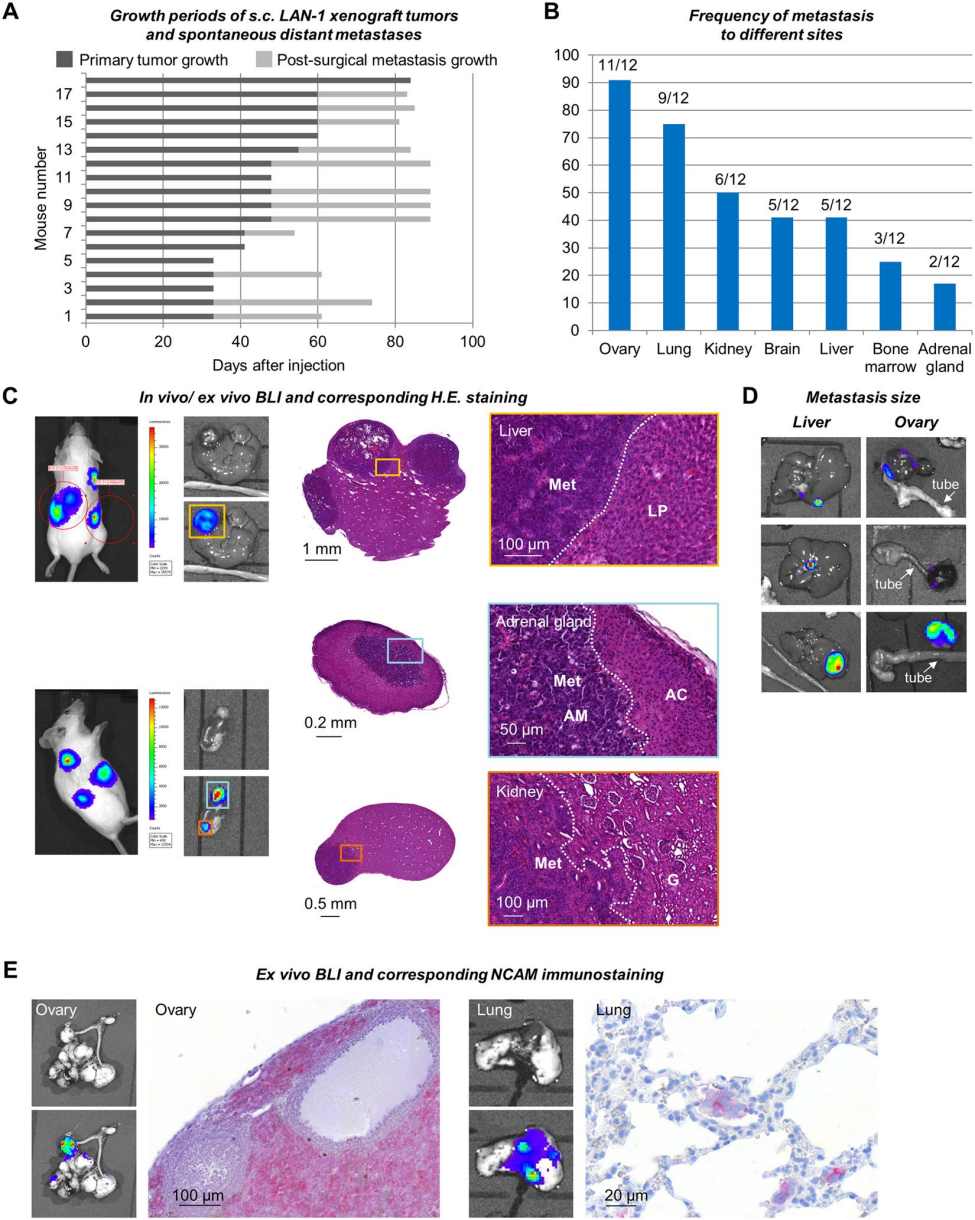
Primary tumor growth and post-operative metastasis patterns. (**A**) Primary tumor and post-operative metastasis growth periods of LAN-1 xeno-engrafted into 18 individual mice. Note that 12 of 18 mice developed distant metastasis detectable by BLI within a maximum of 90 days after injection. (**B**) Frequency of different sites affected by metastasis. (**C**–**E**) Examples of *in vivo* and *ex vivo* bioluminescent images of liver, kidney, adrenal gland, ovarian and lung metastases and corresponding histology. Please note the size differences of metastases at different sites suggesting particularly rapid metastatic outgrowth in the ovaries (**D**). Immunohistochemistry for human NCAM demonstrates the human origin of metastatic lesions (**E**). Met = metastasis; LP = liver parenchyma; AM = adrenal medulla; AC = adrenal cortex; G = glomerulum.

Tumor take rate after s.c. injection of LAN-1-*Luc2/mCherry* cells in Matrigel^TM^ into female *rag2*^*−/−*^ mice was 90% (18 of 20 mice developed tumors). The growth periods until the tumors were about 1.5 cm^3^ in size or started to ulcerate the mouse skin are shown for each individual in Fig. 2A (dark grey bars). At this time point, the mice were scanned by BLI to demonstrate the luminescence of the primary tumors as shown in Fig. 1 (pre-OP). To prolong the overall survival of the mice and by this to enable outgrowth of detectable distant metastases, primary tumors were surgically resected. Post-OP BLI scans were performed to demonstrate the absence of distant metastases at the time of surgery (Fig. 1). The mean weight of excised tumors was 1 ± 0.5 g. Distant metastases were detectable in 66% of mice (12 of 18) after an average of 31.6 ± 9.3 days post-surgery as shown for each individual in Fig. 2A (light grey bars). As soon as a BLI signal was detectable *in vivo*, the respective mouse was sacrificed and all organs were re-scanned *ex vivo* after resection (Fig. 2C,D). Organs with a strong detectable BLI signal were removed from the scanning area to enable detection of smaller metastatic lesions by a third scan. The distribution and frequency of metastasis to different distant sites is shown in Fig. 2B.

All metastatic lesions could be verified by histology by means of morphologic criteria in HE-stainings and by immunostainings against NCAM (neuronal cell adhesion molecule, CD56), which is typically expressed on NB cells. Please see Fig. 2C–E for examples of liver, adrenal gland, kidney, ovarian and lung metastases BLI signals and corresponding histology.

In 11 out of 12 mice ovaries were affected and harbored large, macroscopically visible metastases. Many of these ovarian metastases contained a high proportion of necrotic tissue so that in some cases only a minor part of the metastasis was luminescent (Fig. 2D). Liver, kidney and adrenal gland metastases detectable by BLI and histology were macroscopically smaller than ovarian metastases and only partially visible at necropsy (Fig. 2C,D). As determined by histology, the metastases in the adrenal gland were restricted to the adrenal medulla (Fig. 2C). Lung and brain metastases were only detectable by BLI and histology, but were macroscopically not visible.

### Proteome analysis of IR-laser ablated tissue homogenates

**Figure 3:**
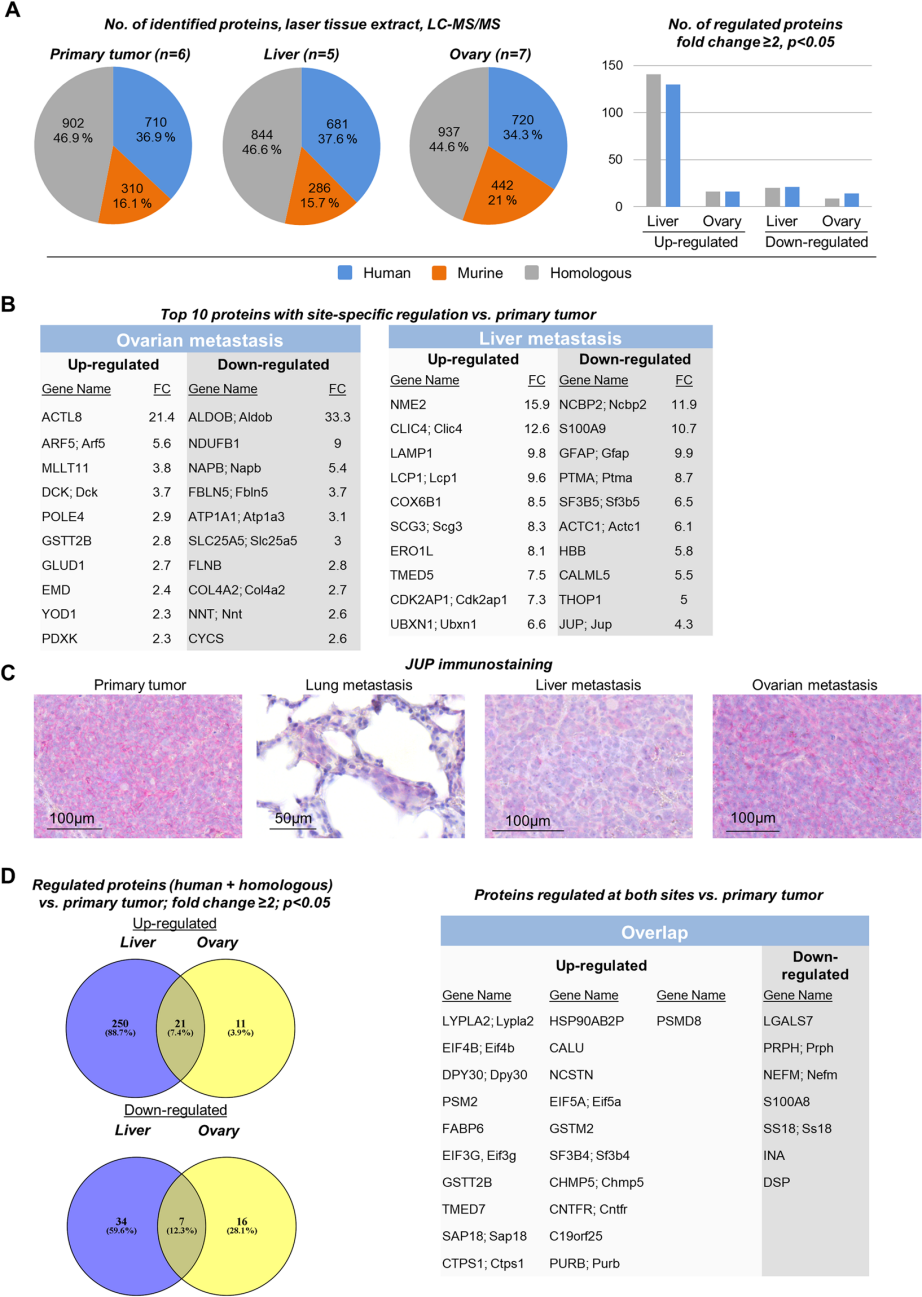
Differential proteome analysis of from primary tumors, liver metastases and ovarian metastases. (**A**) Total number of human, murine and homologous proteins identified by proteome analysis of IR-laser ablated tissue homogenates from primary tumors, liver metastases and ovarian metastases (pie charts on the left); Number of proteins found to be regulated at the different sites (bar chart on the right, discriminating between proteins with human or homologous peptide sequence). (**B**) Gene names of the top 10 proteins up- or down-regulated at both sites. (**C**) Validation of the decreased expression of junctional plakoglobin (JUP) in liver metastases compared to primary tumor as indicated in (**B**). Moreover, JUP was found to be particularly weakly expressed in lung micro-metastases, while large, macroscopically visible ovarian metastases showed strong JUP expression similar to primary xenograft tumors. (**D**) Absolute numbers and gene names of proteins regulated in both ovarian and liver metastases (concordant overlap).

Proteome analysis was performed with tissue ablated with the IR-laser obtained from all biological replicates from primary tumors (n = 18), ovarian metastases (n = 11), and liver metastases (n = 5). After setting the described filters (see above), proteome data from six primary tumors, seven ovarian metastases and five liver metastases were available. The pie charts displayed in Fig. 3A demonstrate the total number and percentage of human, murine and homologous proteins identified in the biological replicates. Among these, 130 homologous and 141 human proteins were significantly up-regulated in liver metastases compared to primary tumors (p < 0.05, fold change ≥2). In contrast, only 21 homologous and 20 human proteins were down-regulated in liver metastases. In ovarian metastases, which were macroscopically larger and more common than liver metastases, only 16 homologous and 16 human proteins were up-regulated while 14 homologous and 9 human proteins were down-regulated (Fig. 3A). Hence, in liver metastases, 20.4% of all identified human and homologous proteins showed differential regulation compared to only 3.3% in ovarian metastases. The top 10 proteins regulated in either ovarian or liver metastases are shown in Fig. 3B (for full lists of all proteins found to be regulated in the *in vivo* samples, please see Supplementry Table S1; for GO annotations please see Supplementry Tables S3–S5). These lists mainly include novel candidates that were so far not or only by a few studies reported to be directly associated with metastases. One example is JUP (junctional plakoglobin), which was down-regulated in liver metastases, but not ovarian metastases, as per proteome analysis analysis (Fig. 3B). Accordingly, immunostainings for JUP revealed strong expression in primary tumors and the large ovarian metastases, but weak expression in the smaller liver and lung micro-metastases (Fig. 3C).

There was an overlap of 21 proteins up-regulated and 7 proteins down-regulated in both ovarian and liver metastases compared to primary tumors as shown in Fig. 3D.

### Functional analysis with LAN-1-PT, LAN-1-Met-O and LAN-1-Met-L sublines

**Figure 4:**
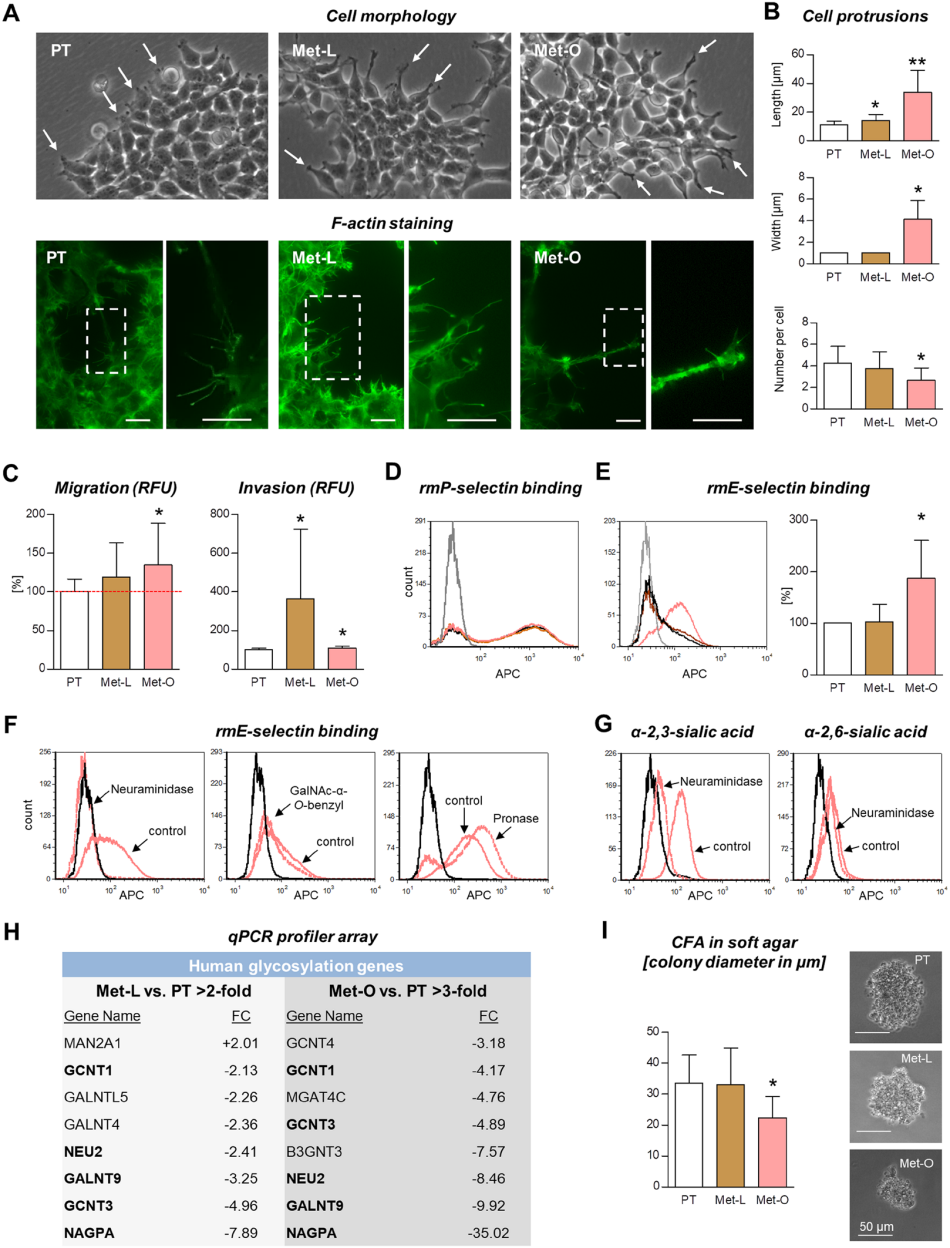
Characterization of *in vitro* sublines. Shortly after recovery from *in situ* specimens, the cells derived from liver (Met-L) and ovarian metastases (Met-O) appeared to have prolonged protrusions (as indicated by light microscopy) compared to the primary tumor subline PT (**A**, arrows). As quantified by F-actin immunocytochemistry, the cell protrusion length and width were markedly increased in Met-O cells, while the number of protrusions per cell was decreased (**B**). Both metastatic cell lines showed increased migratory and/or invasive potential *in vitro* (**C**). P-selectin binding was not affected (**D**) while Met-O cells showed significantly enhanced capacity to bind murine E-selectin (**E**). The E-selectin ligands were completely cleaved from the cell surface by neuraminidase treatment whereas inhibition of *O*-GalNAc-glycosylation (using GalNAc-α-*O*-benzyl) and non-specific cleavage of glycoproteins (using pronase) did not impair, but rather even increased E-selectin binding capability (**F**). The neuraminidase treatment strikingly cleaved α-2,3-linked, but not α-2,6-linked sialic acid residues (**G**). As determined by qPCR profiler arrays, Met-O cells showed stronger gene expression changes in glycosylation enzymes than Met-L cells: the table embedded in (**H**) displays all enzymes regulated in Met-L cells, but only those with >3-fold regulation in Met-O cells. Note that five of the eight enzymes regulated in Met-L cells were more strikingly changed in Met-O cells (concordantly). Colony forming assays in soft agar revealed impaired anchorage-independent growth of Met-O compared to PT cells (**I**). Bar charts represent mean ± SD, *p < 0.05, **p < 0.001. Scale bars in (**A**) represent 10 µm. APC = allophycocyanin; RFU = relative fluorescence units; CFA = colony forming assay.

The sublines of LAN-1 primary tumor (PT), liver metastasis (Met-L) and ovarian metastasis (Met-O), which were retrieved from the *in situ* lesions and expanded *in vitro*, showed morphologic differences as shown in Fig. 4A. Despite the visible prolongation of cellular protrusions in both metastatic sublines compared to the primary tumor (as per light microscopy, arrows), quantification of these protrusions based on F-actin immunocytochemical stainings revealed a significant increase in the protrusion length only in the case of the ovarian metastasis subline, but not the liver metastasis subline (Fig. 4B). Concurrently, these prolonged protrusions of LAN-1-Met-O were significantly wider and less common per cell (Fig. 4B).

*In vitro* assays concerning single steps of the metastatic cascade showed that the ovarian metastasis subline harbored slightly increased migratory potential (134.6 ± 53.7% compared to PT, *p* = 0.044, two independent experiments with six technical replicates each) while the liver metastasis subline showed enhanced invasiveness (with high variability in the single replicates: 363.1 ± 359.4% compared to PT, *p* = 0.018, two independent experiments with six technical replicates each) (Fig. 4C).

One of the next steps of the metastatic cascade, extravasation, requires attachment of CTC to vascular endothelium. This attachment is initiated by the binding of carbohydrate ligands at the tumor cell surface to E- and/or P-selectin at the endothelial surface^6,26^. Therefore, the capacity of the tumor cell lines to bind murine E- and P-selectin was determined next. Interestingly, both metastatic sublines showed no alteration of P-selectin binding capacity (Fig. 4D) while E-selectin binding was enhanced in the ovarian, but not liver metastasis subline (Fig. 4E).

Our biochemical analysis revealed that the increased E-selectin binding of Met-O cells entirely depended on the presence of sialic acid residues as shown by a striking effect of neuraminidase (Fig. 4F). The neuraminidase treatment protocol strongly affected α-2,3-linked sialic acid, whereas α-2,6-linked sialic acid remained nearly unaltered (Fig. 4G). Interestingly, inhbition of *O*-GalNAc-glycosylation as typcially found on mucin-type glycoproteins only marginally reduced E-selectin binding (Fig. 4F). Likewise, even non-specific cleavage of the majority of cell surface glycoproteins using pronase, a mixture of endo- and exopeptidases, did not reduce, but increased murine E-selectin binding of Met-O cells (Fig. 4F). These findings suggest that the increased E-selectin binding of Met-O cells as one pro-metastatic feature might at least in part be due to a different class of biomolecules, namely glycolipids, which are known to bind E-selectin, if they are decorated with terminal α-2,3-linked sialic acid^27^.

In accordance with the particular increase in E-selectin binding of Met-O, but not Met-L cells, glycosyltransferase expression profiler arrays revealed more striking alterations in Met-O than in Met-L cells (compared to PT cells): while Met-L cells showed regulation of only eight enzymes, Met-O cells harbored expression changes of overall 23 glycosyltransferases, of which only those with >3-fold regulation are shown in Fig. 4H. Interestingly, five of the eight regulated enzymes detected im Met-L cells also appeared in the analysis of Met-O cells and were always more stronlgy regulated in the latter ones (Fig. 4H). Those were GCNT1, GCNT3, NEU2, GALNT9 and NAGPA, all of which showed reduced expression compared to PT cells.

The final step in the metastatic process, colonization, was also assessed under three-dimensional conditions *in vitro* using colony formation assays in soft agar. These analyes revealed that the metastatic sublines had no improved capability of forming tumor spheres in soft agar, but even showed decreased colony formation in the case of Met-O cells (Fig. 4I).

### Proteome analysis of cultured cell lines

**Figure 5:**
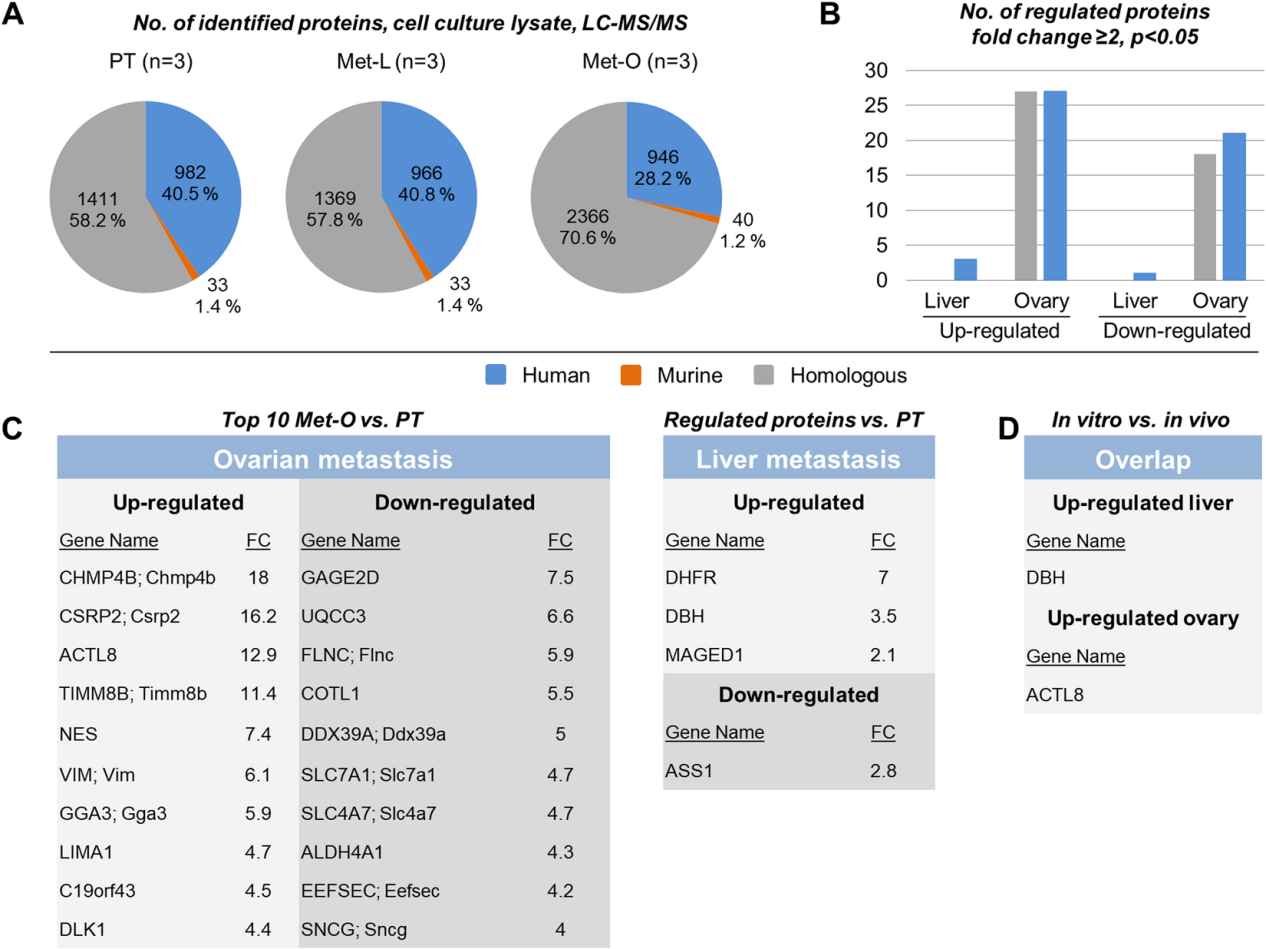
Differential proteome analysis of cultured cells. (**A**) Total number of human, murine and homologous proteins identified by proteome analysis of lysates of cultured cells of primary tumor (PT), liver metastasis (Met-L) and ovarian metastasis (Met-O) sublines. (**B**) Number of proteins found to be regulated at the different sites. The embedded tables in (**C**) display the gene names of proteins found to be up- or down-regulated at both sites (top 10 in the case of Met-O cells, all proteins in the case of Met-L cells). The table in (**D**) shows gene names of proteins that were regulated in both the IR-laser ablated tissue homogenates of *in vivo* specimens and cell lysates of *in vitro* sublines.

LAN-1-PT, LAN-1-Met-O and LAN-1-Met-L samples were subjected to proteome analysis. The pie charts displayed in Fig. 5A demonstrate the total number and percentage of human, murine and homologous proteins identified in all technical replicates. Among all identified proteins, 54 proteins were up-regulated and 39 proteins were down-regulated in the ovarian metastasis subline while 3 proteins were up-regulated and 1 protein was down-regulated in the liver metastasis subline (Fig. 5B). The tables embedded in Fig. 5C display all proteins regulated in the liver metastasis subline and the top 10 proteins regulated in the ovarian metastasis subline (for full lists of all proteins found to be regulated in the *in vitro* samples, please see Supplementry Table S2). Despite the overall low number of regulated proteins, we determined one overlapping protein, dihydrofolate reductase (DHFR), which was up-regulated in both LAN-1-Met-O and LAN-1-Met-L compared to LAN-1-PT.

By comparing the regulated proteins of the *in vivo*-samples (Fig. 3) and the regulated proteins in cell lysates of the *in vitro*-sublines (Fig. 5), we found an overlap of only two proteins (Fig. 5D): the Dopamine Beta-Hydroxylase (DBH) was up-regulated in liver metastasis while the Actin-Like Protein 8 (Cancer/Testis Antigen 57, ACTL8) was up-regulated in ovarian metastasis *in vivo* and *in vitro*.

## Discussion

This is the first study investigating the proteome of spontaneous distant metastases and matched primary tumors in s.c. xenograft mouse models using IR laser tissue extracts. Laser ablation was performed in metastatic tissues detected by BLI so that the whole body was scanned for the presence of metastases *in vivo* and *ex vivo*. Outgrowth of metastases to a detectable size was achieved through surgical resection of the s.c. primary tumors, which is clinically relevant since in many patients metastases become clinically manifest after locally successful therapy of the primary tumor. The post-operative monitoring was mainly shorter than the primary tumor growth period. For this proof-of-principle-study, a highly metastatic cell line was chosen, namely LAN-1 neuroblastoma cells^28^. There are only few studies published on spontaneous metastasis neuroblastoma mouse models that include primary tumor growth^29–31^. Importantly, these studies were mainly based on orthotopic injection of neuroblastoma cells into the adrenal gland since s.c. xenografts have been considered to be non- or weakly metastatic. However, intra-adrenal inoculation is a cumbersome procedure requiring surgery and artificial tumor cell delivery into the circulation during engraftment must be controlled^31^. This artificial dissemination is much more unlikely in s.c. models as the site of injection is less vascularized and the engraftment is performed by injection and no surgical engraftment is required. Moreover, surgical resection of the primary tumor is easily possible in s.c. models and was required in the present study to enable outgrowth of clinically manifest distant metastases. Ultimately, metastases might be derived from other metastases so that the impact of the microenvironment at the primary site might differ among different metastases. For these reasons, we decided to use s.c. xenograft models. Our study demonstrates that at least the s.c. LAN-1 model of human NB is suitable to study metastasis to different sites (ovary > lung > kidney > brain = liver > bone marrow > adrenal medulla).

In general, our unbiased differential proteome analysis revealed a quite short list of regulated proteins suggesting that the distant metastases mainly represent the protein expression patterns of the matched primary tumors. This observation will be discussed below. First, the most strongly regulated proteins, which could be identified, are briefly discussed:

LYPLA2 (APT2), which was up-regulated in both liver and ovarian metastases, is a palmitoyl thioesterase responsible for depalmitoylation of the H- and N-ras GTPases and already under investigation as potential anti-cancer target^32^. The dynamic (de)palmitoylation closely regulates the proper plasma membrane localization, shuttling and recycling of H- and N-ras to the Golgi apparatus and is therefore crucial for the function of these critical regulators of cell fate and cell cycle progression^33^. Our data suggest that the cellular distribution of H- and N-ras regulated by LYPLA2 directly contributes to metastatic progression in our neuroblastoma model. N-ras is an oncogene that has initially been described in neuroblastoma cells^34^.

Interestingly, the downstream signaling cascade of H-/N-ras, the Raf-Mek-Mapk/Erk pathway, activates the eukaryotic translation initiation factor 4b (EIF4B)^35^, which we also found to be strongly up-regulated in both liver and ovarian metastases. All target mRNAs of EIF4B have been reported to control cell proliferation or survival^36^. EIF4B is also activated by PI3K-mTOR signaling so that EIF4B has been concluded to represent a focal point whereby MAPK and mTOR signaling converge^35^. Consequently, EIF4B has been suggested as a promising anti-cancer target^37^. As it appears to be functionally relevant for spontaneous metastasis, EIF4B becomes even more interesting as an anti-cancer target.

Likewise, DPY30, which was found to be strongly up-regulated at both metastatic sites, is an integral core subunit of histone H3K4 methyltransferases. Epigenetic analyses demonstrated that DPY30 directly controls key regulators of cell-cycle progression such as ID proteins and that depletion of DPY30 induces a senescence-like phenotype^38^. Therefore, the up-regulation of DPY30 in the case of the spontaneous metastases in this study might have contributed to the initiation of metastatic colonization by suppressing the senescence/dormancy-like phenotype.

One of the most strongly down-regulated proteins identified at both sites was LGALS7 (Galectin-7). It is widely described that galectin-7 has opposing roles in the context of different cancers and its expression ranges from completely down-regulated to highly up-regulated depending on the tumor type^39^. Interestingly, in the particular case of neuroblastoma, galectin-7 acts as a negative regulator of cell proliferation^40^, which supports our finding of down-regulated expression in metastatic neuroblastoma.

Another example is PRPH (neurofilament 4, 57 kDa), which was found to be down-regulated at both metastatic sites compared to the primary tumors in our neuroblastoma metastasis model. In accordance with this finding, a genome-wide methylation screening study with neuroblastoma patient samples reported that enhanced promotor methylation of the *PRPH* gene (which should result in less *PRPH* expression) is associated with at least one classical risk factor for an unfavorable prognosis (age, stage or MYCN status)^41^. Interestingly, one further candidate among the most strongly down-regulated proteins at both distant sites was NEFM, which is neurofilament 3 (medium polypeptide 150 kDa) and hence belongs to the same protein family as does PRPH. A precise role for NEFM during neuroblastoma progression, invasion or metastasis has not yet been described. Taken together, our findings strongly encourage future studies on the functional implications of repressed neurofilament expression during metastatic progression in neuroblastoma.

312 and 55 proteins were regulated in liver and ovarian metastases, respectively, of which 151 and 30 were homologous proteins and could therefore result from organ-specific protein expression patterns in the murine liver and ovary, respectively. Interestingly, our approach revealed some ‘expectable’ candidates, for which pro-metastatic functions have already been assumed, such as the decrease in desmosomal proteins. Desmosomes are adhesion complexes that might prevent the detachment of single (future metastatic) cells from the primary tumor^42^. Interestingly, three different desmosomal proteins, namely JUP, DSP and DSG1 were found to be down-regulated in liver metastases with JUP being among the top 10 (Fig. 3B) while DSP and DSG1 showed lower fold-changes (Supplementry Table S1). In ovarian metastases, only DSP was found to be down-regulated, but not JUP or DSG1 (Fig. 3D). Other candidates that have been reported to confer pro-metastatic features such as cell adhesion molecules of the immunoglobulin superfamily, integrins, matrix metalloproteinases, etc. have not been identified. The number of proteins found to be regulated in liver metastases was more than 5-fold higher than that in ovarian metastases. This finding might be explained by the differential outgrowth characteristics: while ovarian metastases were frequently large and sometimes even reached the size of the corresponding primary tumor, liver metastases were often notably smaller and less common (Fig. 2D). On the one hand, the large size coincided with a considerable proportion of necrotic areas in ovarian metastases that were not visible by BLI. One could argue that this increased amount of necrotic tissue might have caused a higher degree of protein degradation in ovarian metastases already *in situ*. However, the total number of identifiable proteins was quite similar at all different sites (Fig. 3A), so that this explanation appears unlikely. On the other hand, there is the common hypothesis that large metastases resemble the key characteristics of the primary tumor more closely than small metastases. This hypothesis is based on the assumption that metastasis formation mainly relies on cellular plasticity, implying that the molecular phenotype of a single metastasizing cell or small cell clusters differs much stronger from the primary tumor than that of a large, established metastasis. This assumption would explain why large ovarian metastases show less difference in their protein expression patterns compared to primary tumors than the smaller liver metastases. One widely accepted example for this peculiar cellular plasticity is the induction and reversion of EMT during the metastatic process of solid tumors^43,44^. For instance, tumor cells are supposed to down-regulate epithelial cell junction proteins during EMT at the primary tumor site and to re-express them at the metastatic site during the reversion of EMT, the MET, which takes place during metastatic outgrowth. One example for such epithelial cell junction proteins is the desmosomal protein JUP, which we identified to be down-regulated (among the top 10) in liver (but not ovarian) metastases as per proteome analysis (Fig. 3B). Importantly, as validated by IHC, JUP expression was actually comparatively high in primary tumors and the large ovarian metastases, but low in the smaller liver metastases and particularly weak in the lung micro-metastases (Fig. 3C).

However, not only the differential degree of re-acquisition of primary tumor features in differentially sized metastases might account for the divergent numbers of detectable regulated proteins at different distant sites: another key question is how many of the identified, regulated proteins result from the distinct environmental factors that exist at the different metastatic sites and how many result from the tumor cell’s intrinsic capability to metastasize *per se* (in terms of being biologically determined to be metastatic). The latter ones might largely belong to those proteins that could be found regulated at both sites (overlap, see Fig. 3D) while the former ones are rather those that were found regulated at only one site (see Fig. 3B [top 10] and Supplementry Table S1 [full list]). In the view of the presented data, the influence of environmental factors appears to be site-specific, as the percentage of regulated proteins that were in the overlap between both sites and those found to be regulated at one site varied between the analyzed liver and ovarian metastasis samples (Fig. 3D). Only 8.9% of proteins regulated in liver metastases were also identified in ovarian metastases, but 50.9% of proteins regulated in ovarian metastases were also identified in liver metastases so that the putative impact of environmental factors seemed to be much larger in the liver than in the ovary. Moreover, after *in vitro* expansion of primary tumor and metastasis sublines and subsequent proteome analysis, the number of regulated proteins was much smaller than in IR-laser ablated tissue homogenates (97 vs. 367 proteins) and the overlap between *in vitro* and *in vivo* proteomics was remarkably low (Fig. 5B,D). Therefore, only a very minor proportion of proteins identified to be regulated *in situ* remained persistent *in vitro*. However, it remains unclear whether this is rather due to the lost proximity to the metastatic organ’s milieu or to a general epigenetic reprogramming caused by the cell culture conditions^45^.

Surprisingly, in the case of *in vitro*-cultured sublines, the ovarian metastasis cells harbored a higher number of regulated proteins than the liver metastasis subline, which contrasted the proteomics analysis of the tissue homogenates. In accordance with this discrepancy, the Met-O subline showed more profound phenotypic changes than the Met-L subline (Fig. 4): shortly after recovery from the mice, the ovarian metastasis cells were characterized by notable changes in cellular protrusions, migratory potential, E-selectin binding and glycosyltransferase expression. The increased E-selectin binding capacity of Met-O cells did not depend on *O*-glycosylation, which was also reflected by down-regulation of different glycosyltransferases that initiate *O*-glycan synthesis (GCNT1, GCNT3, GALNT9). Moreover, the E-selectin ligands were concurrently insensitive to pronase treatment suggesting that molecules other than proteins (*e.g*., sialylated glycolipids) mediated the increased E-selectin binding and might therefore have contributed to metastasis formation in our model. Finally, the ovarian metastasis cells showed an impaired colony forming capacity indicating that improved anchorage-independent growth is not essential for metastatic competence (alternatively, this feature could have got lost during the first passages).

Taken together, our study demonstrates that BLI-guided collection and homogenization of metastatic lesions in the LAN-1 neuroblastoma xenograft model is suitable to identify novel candidate proteins that contribute to spontaneous metastasis formation *in vivo*. Moreover, the number of regulated proteins (showing differential expression levels in primary tumors vs. metastatic sites) appears to decrease in advanced metastases. The derived sublines maintain pro-metastatic features *in vitro* making them highly attractive tools for future functional studies.

## Electronic supplementary material

Supplementary Table S1

Supplementary Table S2

Supplementary Table S3

Supplementary Table S4

Supplementary Table S5
